# Behavioral Disorder amongst Adolescents Attending Secondary School in Southeast Nigeria

**DOI:** 10.1155/2014/705835

**Published:** 2014-09-08

**Authors:** J. M. Chinawa, P. C. Manyike, H. A. Obu, O. I. Odetunde, E. C. Aniwada, I. K. Ndu, A. T. Chinawa

**Affiliations:** ^1^College of Medicine, Department of Pediatrics, University of Nigeria and University of Nigeria Teaching Hospital (UNTH), PMB 01129, Ituku-Ozalla, Enugu 400001, Nigeria; ^2^Federal Teaching Hospital, Abakaliki 400001, Nigeria; ^3^College of Community Medicine, University of Nigeria and University of Nigeria Teaching Hospital (UNTH), Ituku-Ozalla, Enugu 400001, Nigeria; ^4^Department of Pediatrics, Enugu State University Teaching Hospital, Enugu 400001, Nigeria; ^5^University of Nigeria and University of Nigeria Teaching Hospital (UNTH), Ituku-Ozalla, Enugu 400001, Nigeria

## Abstract

*Background*. Adolescents are prone to various forms of behavioral problems. These behavioral issues in adolescents can have serious consequences for the adolescents. *Objectives*. The objectives of the study are to determine the causative factors of adolescent problems and specific manifestations. *Methods*. Behavioral problems were investigated using a random sampling of adolescents from secondary schools in southeast Nigeria from February to April, 2014. A self-administered questionnaire was developed from Health Kids Colorado Questionnaire. *Results*. A total of 763 subjects completed the questionnaire. Adolescents who reported to have used tobacco 3 to 5 and 6 to 9 times during the last 30 days are just 3.14% and 3.4%, respectively. Nineteen (2.49%) adolescents claimed that they have had sex before but not in the last 3 months. Adolescents who attempted suicide are from 15 years and peaked at 18. Eighty-three (11%) adolescents who are 15 years old attempted suicide in a year; this peaks at 17 years where 235 (30.8%) committed suicide. Majority of adolescents with behavioral disorder are from the upper class family. *Conclusion*. This study revealed that adolescents exhibit several forms of behavioral problems.

## 1. Introduction

Adolescence (which occurs between 10 and 19 years) is a phase of physical, cognitive, and psychological development that generally occurs during the period from puberty to adulthood [1]. It is pertinent to point out here that adolescent is a “general emotional roller coaster” state of change wherein they become more rational and capable of more complex thinking and tend to evaluate and criticize before arriving at a definite conclusion [2]. It is also a time for developing independence [2, 3].

Adolescent period is a complex maturational and developmental process which varies across individuals and cultures. Successful passage through this portal to adulthood results in biological maturity, a secure sense of self, the ability to enjoy close friendships and group belonging, and the mental capacity to deal with the onslaught of life's challenges [2]. However, failure to manage adequately this physical, emotional, cognitive, and moral unfolding of adolescence can lead to a deviant identity and behavioral anomalies [3].

There are several models to explain behavioral attributes among adolescents. This includes psychodynamic models, behaviorist model, ecological system models, and cognitive models. Indeed, an imbalance between any of the two models can trigger behavioral disorder in adolescents [4]. Furthermore, adolescents are at high risk for the development of behavior clusters that are distressing and socially disruptive [5]. Three clusters were identified, a normal-behavior cluster, a problem-behavior cluster, and a deviant-behavior clusters [5]. Adolescent behavior is found to correlate with events at infancy. For instance, predictive associations existed between parenting and temperament during the first year of life and child conduct problems [6]. Furthermore, infant fussiness correlated well with hyperactivity and conduct problems during ages 4–13 years. In addition, spanking during infancy predicted more severe conduct problems later in adolescents [6]. Bornstein indeed stated the effect of harsh discipline on child aggression. He opined that this aggression is mediated at least in part by maladaptive social information processing patterns that develop in response to the harsh discipline [7]. This is buttressed by McElroy and Rodriguez [8] who noted that several cognitive risk factors significantly predict risk of parental aggression toward children. These include a parent's ability to empathize and take the perspective of their child, parental locus of control, and parental level of frustration tolerance.

Adolescents have varying degree of behavioral problems ranging from violent related behaviors, substance use and misuse, and sexual behaviors to bullying. Each of these problems is not without its burden [9]. For instance, it has been noted that adolescents who misuse drugs such as marijuana develop psychiatric manifestations such as panic stales, flashbacks, drug psychosis, homicides, suicidal thoughts, and drug dependence [9–11].

The problem behaviors in adolescents can have serious consequences for the adolescents, their family and friends, their schools, and society. It has been frequently reported that problem behaviors are the most common reason why adolescents consult psychologist and pediatric psychiatrist [12].

This study is aimed at determining the causative factors of adolescent problems and specific manifestation of behavioral problems among adolescents in Nigeria secondary schools in southeast Nigeria.

In Nigeria, there are very few works on behavioral problems among adolescents in secondary schools. This study, therefore, is among the few to be done in this part of the world; the result will complement earlier studies and will also serve as information base for future researches.

## 2. Methods

### 2.1. Setting

The study was carried out among adolescents in four secondary schools in Enugu and Ebonyi States of Nigeria.

### 2.2. Pretest

A pretest was done using Command Secondary School, Abakpa Nike, Enugu, because of the ethnic and sociocultural diversity of the student population of the school. The aim was to identify and remove ambiguities in the survey instrument.

### 2.3. Instrument Used

Health Kids Colorado Questionnaire (Q) was adapted for this study. The instrument was shown to have satisfactory reliability and validity.

### 2.4. Sampling Technique

#### 2.4.1. School Sampling

Three schools were selected in Enugu and Ebonyi metropolis through stratified random sampling method.

#### 2.4.2. Student Sampling

By a stratified random sampling, a total of 764 students from mixed schools were selected.

The questionnaires which were self-administered were completed by the students under strict examination conditions during class hours after explanation of the purpose of the study. Confidentiality was assured by informing the respondents not to write their names on the questionnaires. The class teachers were excluded from the class during the exercise to avoid their possible influence. Questions arising from the questionnaire were answered by the researcher during the exercise.

Aims are to identify the various types of behavioral problems in adolescents in secondary schools in Enugu and Ebonyi metropolis and their prevalence. This study is also aimed at determining the sociodemographic pattern and circumstances associated with this disorder. Adolescents who gave consent were included in this study while those without consent and those with psychiatric problems were excluded.

The families were assigned to socioeconomic classes using the recommended method (modified) by Warren et al. [13]. The parents' occupation and highest education attained were scored from 1 (the highest) to 5 (the lowest). The mean score for both parents gives social class falling within the 1–5 range. Those with the mean score of <2 were further reclassified into upper class while those with the mean score of >2 were reclassified into lower social class. For the occupation score, those in upper social class included parents, such as senior public officers, large-scale traders, large-scale farmers, and professionals. Lower class included artisans, primary school teachers, peasant farmers, laborers, and the unemployed. For the education score, those with Ph.D., master degree, bachelor degree, and higher national diploma (HND) were categorized as upper class. Those with ordinary national diploma (OND), national certificate of education (NCE), technical education, grade II teachers' certificate, junior and senior secondary school certificate, primary school certificate, and those with no formal education were classified as lower social class [13].

## 3. Ethical Consideration

Consent was specifically approved by the care takers and parents of these children. Written consent was specifically approved by their principals and the students themselves. Participants' consent was also obtained.

### 3.1. Data Analysis

All data were coded, entered, and then analyzed using the Statistical Package for Social Sciences program (SPSS), version 17. Results were presented in cross tabulation and tables. A *P* value less than 0.05 were accepted as significant for each statistical test.

## 4. Results

Table 1 shows the frequency distribution of the respondents by their age, sex, and parental social class. The highest proportion of the respondents was aged 14–16 years comprising 503 (65.8%) followed by those who aged 17–19 years 243 (31.8%) then those who aged 10–13 years 18 (2.4%). Their mean age was 15.87 ± 3.15. Majority of the respondents are males which was 491 (64%) while the remaining 35% were females. The respondents whose parents were in the upper class category were 55.2% while the middle and lower class were almost equal at 22.51% and 22.25% for middle and lower class, respectively.

Table 2 above shows the frequency distribution of the respondents by adolescent problems and specific manifestation of behavioral problems. Adolescents who reported to have not used tobacco were 698 (91.4%) while those who used it for 10 times and above during the last 30 days were 28 (3.7%). Five hundred and eighty-six (76.7%) of them reported to have not taken a prescription drug while 65 (8.5%) has taken for 10 times and above in their life time. Four hundred and sixty-four (60.7%) of the adolescents have not drunk alcohol before while those that started taking alcohol at age of 13 years and above were sixty 184 (24.1%). Most 664 (86.9%) of the respondents claimed they have not been absent from school during the past 30 days; 12 (1.6%) have been absent from school for 4 days and above. The highest proportion 686 (89.79%) of the respondent are of the opinion that finishing school was very important to them while only 3 (0.4%) of them feel that finishing school was not very important to them. Six hundred and forty-two (84.0%) of the adolescents claimed they have never had sex and 86 (11.3%) have had sex but not in the last 3 months, while 7 (0.9%) reported to have had sexual intercourse with 4 or more persons in the last 3 month.

Table 3 shows associations of age in categories with adolescent problems. Over 90% of respondents in all age groups have not taken tobacco while none in 10–13 years, nineteen (3.8%) in 14 to 16 years, and nine (3.7%) in 17–19 years have taken tobacco for 10 days and above in the past 3 months (*P* = 0.243). Higher proportion (204) of those aging 17–19 years (84.0%) have not taken a prescription drug while fourteen (77.8%) and 368 (73.2%) have not taken drug for those aging 10–13 years and 14–16 years, respectively. The difference was significant statistically (*P* = 0.022).

Those aging 17–19 years, 87 (35.8%), have drunk alcohol before for 13 times and above while 95 (18.9%) and 2 (11.1%) have drunk alcohol before for 13 times and above, respectively. The difference was significant statistically (*P* = 0.000). More than 202 (83%) of respondents have never missed school, 5 (1.0%) of those aged 14–19 years, and 9 (2.9%) of those aged 17–19 years have missed school for 4 days and above in the past 30 days (*P* = 0.126).

About 13 (72.2%) of respondents in 10–13 years, 446 (88.7%) in 14 to 16 years, and 227 (93.4%) in 17–19 years said that it is very important to finish high school while none in those aging 10–13 years and 14–19 years and 3 (1.2%) of those aging 17–19 years said that it is not very important. The difference was significant statistically (*P* = 0.001). While 15 (83.3%) of respondents in 10–13 years, 44 (87.5%) in 14 to 16 years, and 187 (77.0%) in 17–19 years have never had sex before, those aging 10–13 years (1 (5.6%)), 14–19 years (5 (1.0%)), and those aging 17–19 years (1 (0.4%)) have had sex with 4 and more persons in the past 3 months. The difference was significant statistically (*P* = 0.000).

Table 4 shows associations of sex with adolescent problems. Over 90% of respondents in both sexes have not taken tobacco while 13 (3.1%) in males and 13 (4.8%) in females have taken tobacco for 10 days and above in the past 3 months (*P* = 0.475). Higher proportions of females 364 (81.3%) have not taken a prescription drug while 222 (74.1%) have not taken drug. Males and females that have taken prescribed drugs were 46 (9.4%) and 19 (7.0%), respectively. The difference was not significant statistically (*P* = 0.079).

About 319 (65.0%) of males and 145 (53.1%) of females have never drunk alcohol before while 105 (21.4%) and 79 (28.9%) have drunk alcohol before for 13 times and above for males and females, respectively. The difference was significant statistically (*P* = 0.006). More than 84% of respondents in both sexes have never missed school because of fear in school or way to school in all age groups while 7 (1.4%) males and 5 (1.8%) females have missed school for 4 days and above in the past 30 days (*P* = 0.374).

Equal proportion (about 90%) of respondents in both males and females said that it is very important to finish high school while none of males and 3 (1.1%) of females said that it is not very important. The difference was not significant statistically (*P* = 0.061). While 430 (87.6%) of males and 212 (77.7%) of females have never had sex before, 2 (0.4%) of males and 5 (1.8%) of females have had sex with 4 and more persons in the past 3 months. The difference was significant statistically (*P* = 0.000).

Table 5 shows associations of social class with adolescent problems. About 90% of respondents in all social classes have not taken tobacco while 15 (3.6%) in upper class, 5 (2.9%) in middle class, and 8 (4.7%) in lower class have taken for 10 days and above in the past 3 months (*P* = 0.367). About 144 (84.0%) in lower and middle class as well as 307 (1.1%) in upper class have not taken a prescription drug while 10.2%, 4.7%, and 8.2% have not taken drug for those in upper, middle, and lower classes, respectively. The difference was significant statistically (*P* = 0.001).

For those in upper class, 79 (18.7%) have drunk alcohol before for 13 times and above while 51 (29.7%) and 54 (31.8%) have drunk alcohol before for 13 times and above, respectively. The difference was significant statistically (*P* = 0.002). About 383 (91%) of respondents in upper class, 138 (80.2%) in middle class, and 143 (84.1%) in lower class have never missed school in all age groups while 0.9% in upper, 2.3% in middle, and 2.4% of those in lower classes have missed school for 4 days and above in the past 30 days. The difference was significant statistically (*P* = 0.008).

About 375 (88.9%) of respondents in upper class, 165 (95.9%) in middle class, and 146 (85.9%) in lower class said that it is very important to finish high school while none of those in middle class, 1 (0.2) for upper class, and 2 (1.2%) for lower class said that it is not very important. The difference was significant statistically (*P* = 0.013). While about 361 (85.0%) of respondents in upper and lower class as well as 136 (79.1%) in middle class have never had sex before, those in upper class (4 (0.9%)), middle class (2(1.2%)), and lower class (1(0.6%)) have had sex with 4 and more persons in the past 3 months. The difference was not significant statistically (*P* = 0.438).

Table 6 shows associations of age in categories with specific manifestation of behavioral problems. Higher proportion of those aging 10–13 years, 2 (11.1%), have thought of suicide in the past 12 months and 30 (6.0%) in 14 to 16 years and 15 (6.2%) in 17–19 years have thought of suicide (*P* = 0.671). Higher proportion of those aging 17–19 years, 53 (21.8%), have been bullied while 3 (16.7%) and 56 (11.1%) have been bullied in those aging 10–13 years and 14–16 years, respectively, in the past 12 months. The difference was significant statistically (*P* = 0.001). Of those aging 14–17 years, 375 (74.6%) have not fought in the past 12 months while 165 (67.9%) and 10 (55.6%) have not fought, respectively. The difference was significant statistically (*P* = 0.008).

Table 7 shows associations of sex with specific manifestation of behavioral problems. Over 65% of respondents in both sexes have not fought while 13 (2.6%) in males and 8 (2.9%) in females have fought for more than 5 times in the past 12 months. The difference was significant statistically (*P* = 0.018). A similar proportion of both males and females (about 6.0%) have thought of committing suicide in the past 12 months. The difference was not significant statistically (*P* = 0.705). About 68 (13.8%) of males and 44 (16.1%) of females admitted that they have been bullied in the past 12 months (*P* = 0.396).

Table 8 shows associations of social class with specific manifestation of behavioral problems. Over 90% of respondents in all social class have had no reason to think of committing suicide in the past 12 months (*P* = 0.514). About 90.0% in upper class, 79.1% in middle class, and 78.8% in lower classes had not been bullied in the past 12 months. The difference was significant statistically (*P* = 0.000). Of those in upper class, 9 (2.1%) have fought for more than 5 times while 8 (4.7%) and 4 (2.4%) have fought for more than 5 times, respectively, in the past 12 months. The difference was not significant statistically (*P* = 0.085).

## 5. Discussion

This study has gone a long way to show that adolescents show variable degrees of behavioral problems. We noted a prevalence of 3.7% among adolescents who use tobacco currently during the last 3 months with majority of them within the age of 14 and 16. This prevalence is similar to the 3.0% recorded in Kano by the NDLEA [14] but less than 6.4% obtained by Friday and colleagues in Calabar, Nigeria [15]. The varied prevalence obtained from this present study and that of Friday could be due to the definition of smoking among the participants in the present study. While the later captured adolescents that smoked in the previous seven days, ours included all adolescents that smoked 30 days (global youth tobacco survey (GYTS)) to the day of data collection. Differences *n* in sample size could also account for these variations in prevalence rates. The use of tobacco in adolescents is reaching troubling levels. It has been reported by World Bank that nearly 82,000–99,000 children and adolescents all over the world begin smoking every day [13]. About half of them would continue to smoke to adulthood and half of the adult smokers are expected to die prematurely due to smoking related diseases [13, 16].

We obtained a prevalence of 24.1% among adolescents who took alcohol especially between the age of 14 and 16. This prevalence is higher than 14.5% obtained by Abdulkarim et al. in Ilorin [17]. This high prevalence could be due to the fact that alcohol is widely available in Nigeria and easily accessible to various age groups. In Nigeria it is also another form of hospitality in the families where it is freely used in the form of beer, palm wine, gin, illicit gin, and “burukutu.”

The prevalence of drug use among adolescents is found to be 8.5% especially between the age of 14 and 16. This is higher than the 3.5% prevalence obtained by NDLEA [14]. The drugs commonly used here are tranquilizers. It is important to note that adolescent students use tranquillizers such as diazepamto induce sleep after using stimulants to keep awake. The current ban on selling such drugs on over the counter will help curb this menace.

We noted in this study that suicide attempt is a behavioral issue among adolescents with a prevalence of 12.5%. Adolescents who attempted suicide are mainly between the ages of 14 and 18. Majority of them are from the upper class. Several reasons such as depression, alcohol, and drug use were adduced as reasons for this.

It has been documented that suicide is the third leading cause of death for adolescents of 15 to 19 years old with a history of depression, a previous suicide attempt, and a family history of psychiatric disorders as notable risk factors [18].

Physical fight and bullying are notable adolescent behavioral trait in this present study. It is more common between the ages of 17 and 19. Interpersonal violence is an important but neglected issue among adolescents. Adolescent physical fighting not only results in injury, disability, and death but is also associated with other potentially harmful behaviors such as substance use and premarital sex [19]. The fact that victims of bullying were more likely to have engaged in physical fighting may be evidence supporting the notion that “violence begets more violence” [20].

Sexual experimentation is a behavioral problem we noted among adolescents in this study though on a small scale. We found that 0.9% of adolescents claimed they have had sex with more than 4 partners in the last 3 months while those who reported to have had sexual intercourse with at least one or more than 2 partners had a prevalence of 3.8%. This prevalence is very low when compared to a study by the National Survey of Adolescent Health (Add Health) where 46 percent of whites and 47 percent Hispanic high school students reported having experiments with sex [21].

It is important to note that children's sexuality develops during early and middle childhood.

Both sexual ideation and activity increase over the adolescent period [22]. Adolescents engage in a spectrum of sexual behaviors ranging from fantasy and self-stimulation to various forms of intercourse [22]. Sexual desires and arousal, sexual experimentation, and the formation of a sexual identity are more pronounced in adolescence [23]. The emerging sexuality that accompanies adolescence poses fundamental challenges. These include adjusting to the altered appearance and functioning of a sexually maturing body, learning to deal with sexual desires, confronting sexual attitudes and values, experimenting sexual behaviors, and integrating these feelings, attitudes, and experiences into a developing sense of self [21]. However, adolescents' responses to these challenges are mainly influenced by the social and cultural context in which they live [22]. For instance, in Nigeria, it is a taboo and abominable for youths to experiment both coital and noncoital sex before marriage. This may account for the low prevalence we had in this study. Another reason for this low prevalence of sexual experimentation among adolescents in this study could be due to underreporting. The poorly reported prevalence could be due to the fact that in this environment it is stigmatizing and dehumanizing to report cases of sexual abuse.

School absenteeism is a form of behavioral problem noted among adolescents. We found that a fraction of adolescents (1.6%) reported to have been absent from school for more than twice in a week while less than one percent do so up to one week in a month. This is very low when compared to the study of Ananthakrishnan and Nalini [23] in India who found that 30 out of 54 adolescent girls and 40 out of 89 adolescent boys had absented from school on one or more occasions. The reason why we obtain a low value in this study is because most of the schools in this setting have a very good school health program. This will always stimulate the students and lure them to always be in school.

When we looked at gender with its association with substance use as a behavioral issue among adolescent, we noted that males tend to use tobacco, alcohol, and even drugs more than females. Most of literatures reported high male preponderance in all substance use among adolescents. However, more females reported being exposed to sex and admitted that finishing school is not important when compared to their male folks. There are salient gender differences in the psychological impact of variations in the timing of puberty; for instance, early pubertal development in girls is correlated with lower self-esteem and heightened concern over body image [24]. This low esteem and poor body image may tilt the female adolescents to resort to sexual experimentation. From this study, behavioral problems are not common at early adolescents (from ten to thirteen) but become manifest from the age of 14 to 16 and wanes thereafter. During the earlier phase of adolescence, a heightened sense of grandiosity and invulnerability is merged with a more limited capacity to anticipate immediate danger and to foresee long-term negative consequences. This risk potential may be increased as adolescent progresses in age but peaks at very late adolescent. This may account for the experimentation of and involvement in sexual activity, use of alcohol or other drugs, and courting of danger [24]. Moreover, there are major changes in brain structure from childhood through the early adult years [24]. These findings provide a basis for understanding differences between adolescent and adult thought processing. For example, the ventromedial prefrontal cortex is known to be associated with the ability to estimate risk and to guide decision making. Imaging studies show that this brain region is among the last to develop fully. This may also explain the risk-taking behavior during this mid-adolescence [24].

We noted with interest a trend when we correlate the social class of the parents of adolescents with adolescent behaviors. Majority of the parents of adolescents with all forms of behavioral problems are from a high socioeconomic class. In fact, adolescents who have the least behavioral problems have parents from a lower socioeconomic class. There are conflicting reports on the impact of socioeconomic class on adolescent behavior. For instance, Petridou et al. [25] opined that high-risk behavior is mostly among adolescents from the less privileged families and population group. Lahey et al. [12] in their study also noted that children living in lower socioeconomic environments are more likely to develop conduct problems than children living in higher socioeconomic environments. In the contrary, Vuković and Bjegović-Mikanović [26] in their work highlighted that adolescents with higher weekly income and whose parents are from a high social class are more likely to ever have sex, use contraception, and indulge in all sort of behavioral problems than their counterparts with less weekly disposable money. The schools selected for this study are located in the city where parents of high social class cluster. A study of this nature in a wider population and in a rural or suburban population may help solve these controversies.

## 6. Conclusion

This study revealed that adolescents exhibit several forms of behavioral problems. However, a longitudinal study is necessary to determine the changing pattern of this disorder.

### 6.1. Limitations

The limitation of this study lies in the fact that a cross-sectional survey was done. A longitudinal study would have permitted the evaluation of the changing pattern of behavioral problems over time.

## Figures and Tables

**Table 1:** Sociodemographic characteristics of the respondents.

Variable	Frequency (*n* = 764)	Percent (100)
Age		
10–13	18	2.4
14–16	503	65.8
17–19	243	31.8
Mean ± SD	**15.87 ± 3.15**	
Sex		
Male	491	64.3
Female	273	35.7
Social class		
Upper class	422	55.2
Middle class	172	22.5
Lower class	170	22.3

**Table 2:** Frequency distribution of the respondents by adolescent problems and specific manifestation of behavioral problems.

Variable	Frequency (*n* = 764)	Percent (100)
Number of times you had tobacco (cigarette) in past 3 months		
0 days	698	91.4
9 days and below	38	5.0
10 days and above	28	3.7
Number of times you have taken a prescription drug in your life		
0 times	586	76.7
1 to 9 times	113	14.8
10 times and above	65	8.5
Duration of not going to school the past 30 days because of your safety in school or on your way to school		
0 days	664	86.9
3 days and below	88	11.5
4 days and above	12	1.6
Importance of finishing high school to you		
Very important	686	89.8
Important	75	9.8
Not very important	3	0.4
Number of persons you had sexual intercourse with in past 3 months		
Never had sexual intercourse	642	84.0
Had sexual intercourse, but not in the last 3 months	86	11.3
1–3 persons	29	3.8
4 persons and above	7	0.9

**Table 3:** Associations of age in categories with adolescent problems.

	Number of times you had tobacco (cigarette) in past 3 months			
	None	≤9	≥10	
Age (Years)				
10–13	17 (94.4)	1 (5.6)	0 (0.0)	
14–16	453 (90.1)	31 (6.2)	19 (3.8)	
17–19	228 (93.8)	6 (2.5)	9 (3.7)	
	**χ** ^2^ = 5.459		**P** ** = 0.243**	

	Number of times you have taken a prescription drug in your life			
	None	1–9	≥10	

Age (Years)				
10–13	14 (77.8)	2 (11.1)	2 (11.1)	
14–16	368 (73.2)	84 (16.7)	51 (10.1)	
17–19	204 (84.0)	27 (11.1)	12 (4.9)	
	**χ** ^2^ = 11.474		**P** ** = 0.022**	

	Age at first drink of alcohol other than a few sips			
	None	<13	≥13	

Age (Years)				
10–13	9 (50.0)	7 (38.9)	2 (11.1)	
14–16	337 (67.0)	71 (14.1)	95 (18.9)	
17–19	118 (48.6)	38 (15.6)	87 (35.8)	
	**χ** ^2^ = 37.349		**P** ** = 0.000**	

	Duration of not going to school the past 30 days because of your safety in school or on your way to school			
	None	≤3	≥4	

Age (Years)				
10–13	15 (83.3)	3 (16.7)	0 (0.0)	
14–16	447 (88.9)	51 (10.1)	5 (1.0)	
17–19	202 (83.1)	34 (14.0)	7 (2.9)	
	**χ** ^2^ = 7.186		**P** ** = 0.126**	

	Importance of finishing high school to you			
	Very important	Important	Not very important	

Age (Years)				
10–13	13 (72.2)	5 (27.8)	0 (0.0)	
14–16	446 (88.7)	57 (11.3)	0 (0.0)	
17–19	227 (93.4)	13 (5.3)	3 (1.2)	
	**χ** ^2^ = 19.508		**P** ** = 0.001**	

	Number of persons you had sexual intercourse with in past 3 months			
	None	Not in last 3 months	1–3	≥4

Age (Years)				
10–13	15 (83.3)	1 (5.6)	1 (5.6)	1 (5.6)
14–16	440 (87.5)	48 (9.5)	10 (2.0)	5 (1.0)
17–19	187 (77.0)	37 (15.2)	18 (7.4)	1 (0.4)
	**χ** ^2^ = 25.158		**P** ** = 0.000**	

**Table 4:** Associations of sex with adolescent problems.

	Number of times you had tobacco (cigarette) in past 3 months			
	None	≤9	≥10	
Sex				
Male	452 (92.1)	24 (4.9)	15 (3.1)	
Female	246 (90.1)	14 (5.1)	13 (4.8)	
	**χ** ^2^ = 1.488		**P** ** = 0.475**	

	Number of times you have taken a prescription drug in your life			
	None	1–9	≥10	

Sex				
Male	364 (74.1)	81 (16.5)	46 (9.4)	
Female	222 (81.3)	32 (11.7)	19 (7.0)	
	**χ** ^2^ = 5.082		**P** ** = 0.079**	

	Age at first drink of alcohol other than a few sips			
	None	<13	≥13	

Sex				
Male	319 (65.0)	67 (13.6)	105 (21.4)	
Female	145 (53.1)	49 (17.9)	79 (28.9)	
	**χ** ^2^ = 10.356		**P** ** = 0.006**	

	Duration of not going to school the past 30 days because of your safety in school or on your way to school			
	None	≤3	≥4	

Sex				
Male	433 (88.2)	51 (10.4)	7 (1.4)	
Female	231 (84.6)	37 (13.6)	5 (1.8)	
	**χ** ^2^ = 1.968		**P** = **0.374**	

	Importance of finishing high school to you			
	Very important	Important	Not very important	

Sex				
Male	441 (89.8)	50 (10.2)	0 (0.0)	
Female	245 (89.7)	25 (9.2)	3 (1.1)	
	**χ** ^2^ = 5.584		**P** ** = 0.061**	

	Number of persons you had sexual intercourse with in past 3 months			
	None	Not in last 3 months	1–3	≥4

Sex				
Male	430 (87.6)	49 (10.0)	10 (2.0)	2 (0.4)
Female	212 (77.7)	37 (13.6)	19 (7.0)	5 (1.8)
	**χ** ^2^ = 19.132		**P** ** = 0.000**	

**Table 5:** Associations of social class with adolescent problems.

	Number of times you had tobacco (cigarette) in past 3 months			
	None	≤9	≥10	
Social class				
Upper class	382 (90.5)	25 (5.9)	15 (3.6)	
Middle class	163 (94.8)	4 (2.3)	5 (2.9)	
Lower class	153 (90.0)	9 (5.3)	8 (4.7)	
	**χ** ^2^ = 4.298		**P** ** = 0.367**	

	Number of times you have taken a prescription drug in your life			
	None	1–9	≥10	

Social class				
Upper class	300 (71.1)	79 (18.7)	43 (10.2)	
Middle class	144 (83.7)	20 (11.6)	8 (4.7)	
Lower class	142 (83.5)	14 (8.2)	14 (8.2)	
	**χ** ^2^ = 18.804	**P** ** = 0.001**		

	Age at first drink of alcohol other than a few sips			
	None	<13	≥13	

Social class				
Upper class	281 (66.6)	62 (14.7)	79 (18.7)	
Middle class	91 (52.9)	30 (17.4)	51 (29.7)	
Lower class	92 (54.1)	24 (14.1)	54 (31.8)	
	**χ** ^2^ = 17.532	**P** ** = 0.002**	

	Duration of not going to school the past 30 days because of your safety in school or on your way to school			
	none	≤3	≥4	

Social class				
Upper class	383 (90.8)	35 (8.3)	4 (0.9)	
Middle class	138 (80.2)	30 (17.4)	4 (2.3)	
Lower class	143 (84.1)	23 (13.5)	4 (2.4)	
	**χ** ^2^ = 13.728	**P** ** = 0.008**		

	Importance of finishing high school to you			
	Very important	Important	Not very important	

Social class				
Upper class	375 (88.9)	46 (10.9)	1 (0.2)	
Middle class	165 (95.9)	7 (4.1)	0 (0.0)	
Lower class	146 (85.9)	22 (12.9)	2 (1.2)	
	**χ** ^2^ = 12.630	**P** ** = 0.013**		

	Number of persons you had sexual intercourse with in past 3 months			
	None	Not in last 3 months	1–3	≥4

Social class				
Upper class	361 (85.5)	44 (10.4)	13 (3.1)	4 (0.9)
Middle class	136 (79.1)	23 (13.4)	11 (6.4)	2 (1.2)
Lower class	145 (85.3)	19 (11.2)	5 (2.9)	1 (0.6)
	**χ** ^2^ = 5.871	**P** ** = 0.438**		

**Table 6:** Associations of age in categories with specific manifestation of behavioral problems.

	Number of times of physical fight in past 12 months		
	Never	1–5	>5
Age (Years)			
10–13	10 (55.6)	8 (44.4)	**0 (0.0)**
14–16	375 (74.6)	120 (23.9)	**8 (1.6)**
17–19	165 (67.9)	65 (26.7)	**13 (5.3)**
	**χ** ^2^ = 13.857	**P** ** = 0.008**	

	Ever thought of suicide in past 12 months		
	Yes	No	

Age (Years)			
10–13	2 (11.1)	16 (88.9)	
14–16	30 (6.0)	473 (94.0)	
17–19	15 (6.2)	228 (93.8)	
	**χ** ^2^ = 0.798	**P** ** = 0.671**	

	Ever bullied in past 12 months		
	Yes	No	

Age (Years)			
10–13	3 (16.7)	15 (83.3)	
14–16	56 (11.1)	447 (88.9)	
17–19	53 (21.8)	190 (78.2)	
	**χ** ^2^ = 14.991	**P** ** = 0.001**	

**Table 7:** Associations of sex with specific manifestation of behavioral problems.

	Number of times of physical fight in past 12 months		
	Never	1–5	>5
Sex			
Male	370 (75.4)	108 (22.0)	13 (2.6)
Female	180 (65.9)	85 (31.1)	8 (2.9)
	**χ** ^2^ = 8.016	**P** ** = 0.018**	

	Ever thought of suicide in past 12 months		
	Yes	No	

Sex			
Male	29 (5.9)	462 (94.1)	
Female	18 (6.6)	255 (93.4)	
	**χ** ^2^ = 0.143	**P** ** = 0.705**	

	Ever bullied in past 12 months		
	Yes	No	

Sex			
Male	68 (13.8)	423 (86.2)	
Female	44 (16.1)	229 (83.9)	
	**χ** ^2^ = 0.721	**P** ** = 0.396**	

**Table 8:** Associations of social class with specific manifestation of behavioral problems.

	Number of times of physical fight in past 12 months		
	None	1–5	>5
Social class			
Upper class	318 (75.4)	95 (22.5)	9 (2.1)
Middle class	119 (69.2)	45 (26.2)	8 (4.7)
Lower class	113 (66.5)	53 (31.2)	4 (2.4)
	**χ** ^2^ = 8.188	**P** ** = 0.085**	

	Ever thought of suicide in past 12 months		
	Yes	No	

Social class			
Upper class	26 (6.2)	396 (93.8)	
Middle class	8 (4.7)	164 (95.3)	
Lower class	13 (7.6)	157 (92.4)	
	**χ** ^2^ = 1.329	**P** ** = 0.514**	

	Ever bullied in past 12 months		
	Yes	No	

Social class			
Upper class	40 (9.5)	382 (90.5)	
Middle class	36 (20.9)	136 (79.1)	
Lower class	36 (21.2)	134 (78.8)	
	**χ** ^2^ = 20.231	**P** ** = 0.000**	
